# Investigation of Self-Powered IoT Sensor Nodes for Harvesting Hybrid Indoor Ambient Light and Heat Energy

**DOI:** 10.3390/s23083796

**Published:** 2023-04-07

**Authors:** Heng Xiao, Nanjian Qi, Yajiang Yin, Shijie Yu, Xiangzheng Sun, Guozhe Xuan, Jie Liu, Shanpeng Xiao, Yuan Li, Yizheng Li

**Affiliations:** 1 School of Instrument Science and Opto-Electronics Engineering, Beijing Information Science and Technology University, Beijing 100192, China; 2 Department of Precision Instrument, Tsinghua University, Beijing 100084, China; 3 State Key Laboratory of Precision Measurement Technology and Instruments, Tsinghua University, Beijing 100084, China; 4 Beijing Advanced Innovation Center for Integrated Circuits, Beijing 100084, China; 5 China Mobile Research Institute, Beijing 100053, China

**Keywords:** hybrid energy harvesting, Internet of Things (IoT), self-powered, solar energy, thermoelectric

## Abstract

Sensor nodes are critical components of the Internet of Things (IoT). Traditional IoT sensor nodes are typically powered by disposable batteries, making it difficult to meet the requirements for long lifetime, miniaturization, and zero maintenance. Hybrid energy systems that integrate energy harvesting, storage, and management are expected to provide a new power source for IoT sensor nodes. This research describes an integrated cube-shaped photovoltaic (PV) and thermal hybrid energy-harvesting system that can be utilized to power IoT sensor nodes with active RFID tags. The indoor light energy was harvested using 5-sided PV cells, which could generate 3 times more energy than most current studies using single-sided PV cells. In addition, two vertically stacked thermoelectrical generators (TEG) with a heat sink were utilized to harvest thermal energy. Compared to one TEG, the harvested power was improved by more than 219.48%. In addition, an energy management module with a semi-active configuration was designed to manage the energy stored by the Li-ion battery and supercapacitor (SC). Finally, the system was integrated into a 44 mm × 44 mm × 40 mm cube. The experimental results showed that the system was able to generate a power output of 192.48 µW using indoor ambient light and the heat from a computer adapter. Furthermore, the system was capable of providing stable and continuous power for an IoT sensor node used for monitoring indoor temperature over a prolonged period.

## 1. Introduction

With the rapid development of sensor and microelectromechanical technologies, the performance of IoT sensor devices has increased and their size has shrunk [1]. As a result, these devices have garnered tremendous attention from both industry and the public in the past decade, owing to their versatility and applicability in numerous domains such as smart logistics, smart factories, smart agriculture, and smart home-building [2].

However, IoT sensors powered by batteries have a limited lifespan and maintaining them can be costly. Moreover, discarded batteries containing various heavy metals pose a significant environmental burden [3]. In this context, harvesting energy from the environment has emerged as a promising alternative to batteries, attracting interest from researchers across various fields. By harnessing energy from sources such as light, heat, and mechanical energy, the IoT system’s sensors can enjoy significantly extended lifespans [4,5,6,7,8].

Although the environment contains numerous energy sources, the energy is typically weak and scattered, especially in indoor situations where the use of sensor nodes is of great importance (e.g., smart homes, smart offices, and smart buildings) [9]. Since environmental monitoring parameters (e.g., temperature, humidity, CO_2_ concentration, etc.) do not fluctuate frequently, IoT nodes only need to operate intermittently in active mode for sensing, processing, communication, etc. This sleep-active mode significantly reduces the average power consumption of sensor nodes [10]. On the other hand, hybrid energy-harvesting systems that can harvest two or more types of ambient energy can provide a stable power source to accommodate complex applications and unpredictable environments [11]. In this study, thermal energy, the power density of which is comparable to that of indoor light energy, was chosen as an indoor energy source combined with light energy. Thermal energy is abundant in the environment, including body heat, waste heat from high-powered home appliances, and heat from industrial machines. In many cases, thermal energy is continuously available and can power the system in the absence of light (e.g., at midnight when there is no sunlight or fluorescent light) [12].

Researchers have developed many self-powered IoT nodes for both outdoor and indoor applications, typically including an energy harvester, energy management circuitry, and a wireless sensor node [13,14]. Solar power is one of the most abundant and preferred sources of energy for charging batteries due to its ubiquitous presence [15]. The use of small-sized photocells as an embedded power source for microsensors is critical. Yue et al. proposed an indoor light energy-harvesting system for building air quality sensing, with dimensions of 50 mm × 20 mm × 15 mm. To demonstrate and validate the system, it was used to power an IoT sensor for CO_2_ concentrations [16]. Magno et al. developed a wearable watch bracelet that harvested human body heat and solar energy, using thermoelectric generators and PV cells installed on the front and back of the wristband. The harvested energy was stored in lithium batteries to power low-power cameras, microphones, and other devices [17]. Mishu et al. presented a hybrid thermoelectric-photovoltaic energy-harvesting system for self-powered IoT sensor applications that could generate 0.14 W of power with a minimum of 50 lux of indoor lighting and minimum temperature differential of 5 °C [18]. Previous research on solar energy harvesting has used top-mounted PV cells, which do not fully utilize the solar energy available in the environment. Yang et al. proposed a self-powered microsystem for vibration detection and target recognition that harvested solar energy from the environment and stored it in a lithium battery to power the microcontroller unit and sensors. The microsystem used five-sided PV cells to maximize the harvested solar energy for the system’s size [19]. This method of harvesting energy by placing PV cells on each of the system’s five surfaces is also highly suitable for indoor environments, but currently, there is a lack of relevant research.

The recovery of waste heat is a promising approach, which can be achieved using the Seebeck effect. A TEG can be employed to produce electricity at the interfaces where steep temperature gradients exist in close proximity [20]. Prijic et al. implemented the power supply of a temperature measurement sensor using a commercial TEG. To improve the temperature gradient of the TEG, an aluminum core printed circuit board (PCB) was used as an efficient heat collector and heat sink [21]. Xia et al. developed an ultra-low input power management system for harvesting human body heat to power wireless sensors. In this case, three TEGs were vertically stacked to harvest energy with a lower temperature difference and recover waste heat to a greater extent [22]. Multiple TEGs in series are more versatile than other ways to improve heat harvesting. Additionally, hybrid energy storage technologies that have emerged in recent years are expected to be applied to sensor nodes in order to improve battery life and other energy storage unit performance.

Ambient energy often exhibits noncontinuous behavior and power output can be limited. Therefore, energy storage systems must be used to store excess energy when more is harvested than consumed. However, research on implementing hybrid energy storage systems in wireless sensor network (WSN) nodes is still in its infancy [23,24,25]. Li et al. proposed a multi energy-harvesting device that could simultaneously harvest solar and vibration energy. The energy storage devices were selected from SCs and lithium batteries, and the hybrid energy storage configuration was simple and efficient with a passive parallel topology. However, the power distribution of the system was determined by the internal resistance parameters of the batteries and SCs themselves, which are not externally controlled, so the battery may not have been sufficiently protected [26]. Qi et al. first proposed the novel two-port hybrid diode semi-active topology in the WSN node area and concluded that the configuration had less energy loss, cost, size, and control complexity than the conventional semi-active configuration, making it suitable for small WSN nodes [27]. The existing environmental energy-harvesting systems pay relatively little attention to hybrid energy storage subsystems.

Table 1 summarizes a comparison of different hybrid energy-harvesting systems. The current indoor light energy-harvesting systems have limited energy-harvesting capabilities, relying solely on top-mounted PVs. Thus, the surface area of these systems has not been fully exploited to harvest indoor light energy. On the other hand, self-powered sensing nodes operating on thermal energy are usually equipped with a single TEG to harvest energy. However, the small open-circuit voltage of a single TEG is not ideal for energy-harvesting circuit design. Furthermore, only a handful of energy-harvesting systems integrate hybrid energy storage subsystems, and little attention has been paid to the system integration of self-powered IoT nodes. In the next generation of IoT sensor nodes, there will be a strong emphasis on the system integration of self-powered nodes to overcome these challenges. In response to the aforementioned limitations, we have made the following contributions:

1. The system has been equipped with PV cells on five light-receiving surfaces to improve its capacity to harvest indoor light energy.
2. A TEG device consisting of two TEGs and a heat sink has been incorporated into the system to harvest heat energy. The electrical performance between the TEGs is connected in series to enhance the open-circuit voltage of the TEG device. Furthermore, a hybrid energy-harvesting system has been implemented to address the limitations of a single energy harvester.
3. An energy management module has been integrated into the system to manage energy resources in lithium batteries and supercapacitors more efficiently. This will allow for more efficient energy utilization, reduce waste, and extend the overall lifespan of the system.

This paper describes the development of an ambient source-based hybrid energy-harvesting system (HEHS) that can provide continuous power to IoT sensor nodes. The system combined small solar PV cells and TEGs to harvest ambient energy from both light and thermal sources. To ensure reliable and efficient operation, we also designed an energy storage module with a hybrid diode semi-active topology and modified SC first energy management strategy (EMS). The hybrid energy storage approach allowed the SC to supply the high-power pulse demands of the sensor node, while the lithium battery provided indirect power to the system, thus avoiding high-current discharge and fully leveraging the high-power density of the SC and high energy density of the lithium battery. Finally, we demonstrated the potential of our self-powered IoT sensor node by implementing an indoor temperature monitoring and early warning system.

## 2. System Architecture and Components

Table 2 displays the power densities of various indoor energy sources, highlighting the relatively low power output of artificial lighting sources under indoor illumination. For example, a solar cell’s power density in an indoor environment with irradiance is about 0.1 mW/cm^2^, which is significantly lower than the 100 mW/cm^2^ output in normal outdoor monitoring conditions. The total capacity of all indoor artificial energy sources is also smaller than that of outdoor sources [28]. To ensure continuous operation of sensor nodes in indoor environments, it is necessary to add alternative energy sources. In this article, we proposed self-powered IoT sensor nodes that could harvest energy from both thermal and light sources. Light and thermal energies in indoor environments have slightly different power densities. Additionally, waste heat generated by continuous machines or powerful home appliances can be used to supplement intermittent light energy in offices, hospitals, or factories. When ambient energy is unavailable, energy storage devices can be utilized to power the system.

**Figure 1:**
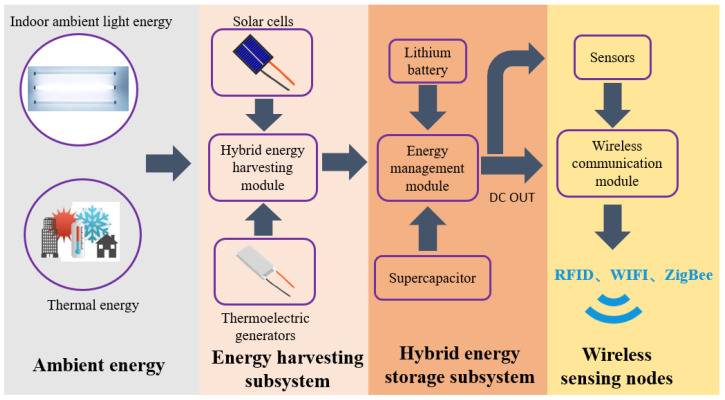
System framework of the proposed self-powered IoT sensor node.

Figure 1 illustrates the system framework of the proposed self-powered IoT sensor node. The system comprised an ambient energy-harvesting subsystem, a hybrid energy storage subsystem, and a wireless sensor node. The ambient energy-harvesting subsystem consisted of energy harvesters, namely, PV cells and TEGs, and a hybrid energy-harvesting module. The hybrid energy storage subsystem comprised energy storage devices, specifically a lithium battery and SC, and an energy management module.

When subjected to indoor ambient energy excitation (e.g., indoor artificial light sources, natural light filtered through windows, waste heat from factory machines), both PV cells and TEGs generate direct current (DC) power, albeit with different electrical characteristics. A single PV cell or TEG cannot satisfy the power demand of a wireless sensor node, thereby necessitating the use of hybrid strategies based on two or more mechanisms [30]. The energy-harvesting subsystem ensured optimal power generation performance of the harvesting device, while the hybrid energy storage subsystem stored the intermittent power generated by the energy-harvesting subsystem in a lithium battery, which powered the IoT sensor nodes. The introduction of solar and thermal energy eliminates the need for frequent battery replacements, minimizes environment pollution, prolongs the system’s lifetime, and lowers system maintenance costs.

In addition, the system utilized 2.4 GHz active RFID communication, which boasts a superior communication range and greater flexibility compared to traditional passive RFID communication. The latter relies on a customized and intricate reader to receive and power the signal, limiting its applicability. Conversely, the active RFID technology employed by the proposed system enables the exchange of customized information, thus allowing for a wide range of potential applications, including building health monitoring, early warning systems, wireless indoor positioning, and other uses.

## 3. Circuit and Integration Design of the System

### 3.1. Energy-Harvesting Subsystem

The energy-harvesting subsystem converted unstable light and thermal energy into electrical energy that directly powered the sensor nodes. The subsystem was comprised of a solar energy-harvesting circuit, thermal energy-harvesting circuit, and energy harvesters.

#### 3.1.1. Solar Energy-Harvesting Circuit

**Figure 2:**
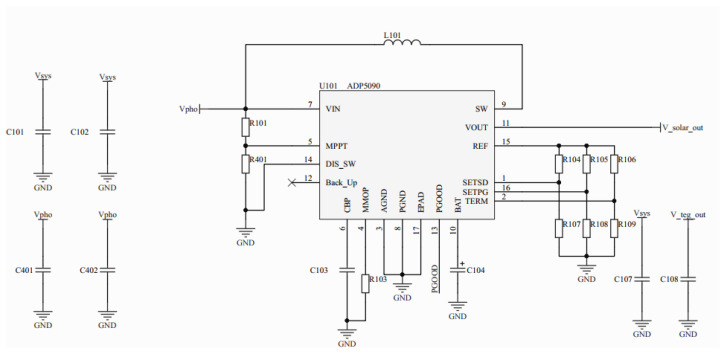
Scheme of the solar energy-harvesting circuit.

The solar energy-harvesting circuit, as illustrated in Figure 2, was centered around the ADP5090 ultra-low power chip, which integrates a nanometer power boost regulator and an energy storage element management controller. The circuit was designed to convert DC power from PV sources and was capable of charging both a rechargeable battery and an SC. To achieve this, each of the system’s five light-receiving surfaces was covered with a 40 mm × 40 mm × 1.1 mm amorphous silicon solar cell, which were then connected in parallel to the input interface of the solar energy-harvesting circuit. Amorphous silicon solar cells were chosen as light harvesters because they have a higher conversion efficiency than crystalline silicon solar cells in low-light indoor conditions [31]. Although there was a significant difference in light irradiance across the five surfaces of the system, especially in indoor artificial light, the output voltage of each amorphous silicon solar cell was only slightly affected by light irradiance. Therefore, each PV side could be connected in parallel without significant impact on their output power. Consequently, the solar energy could be efficiently harvested by the system, and the circuit was capable of outputting a steady 4.2 V.

#### 3.1.2. Thermal Energy-Harvesting Circuit

**Figure 3:**
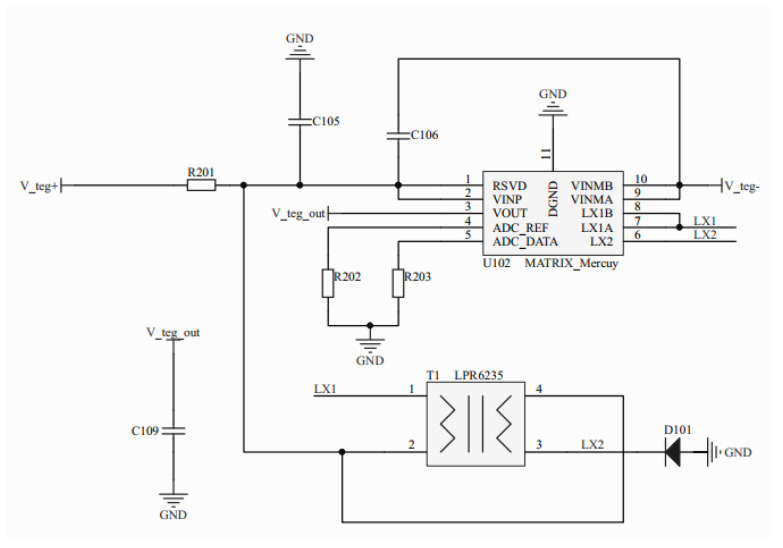
Scheme of the thermal energy-harvesting circuit.

The MCRY12-125Q-46WI served as the central component in the thermal energy-harvesting circuit, facilitating efficient energy harvesting from the TEG. The thermal energy-harvesting circuit is illustrated in Figure 3. This integrated circuit came equipped with an input impedance matching function that ensured the TEG was always in an optimal state for energy harvesting. The circuit was designed to transform DC power from a commercial TEG made of Bi_2_Te_3_ (bismuth telluride) thermoelectric material. This TEG had a physical size of 40 mm × 40 mm × 4 mm and an internal resistance of 6 Ω. To match the circuit’s 12 Ω input impedance, two TEGs were linked in series. The TEG harnessed thermal energy and converted it into electricity through the Seebeck effect [32], which can be expressed by Equation (1):

(1) $$U_{oc}=\alpha \times \left(T_{h}-T_{c}\right)$$

where $U_{oc}$ is the open-circuit voltage of the TEG, α is the Seebeck coefficient of the TEG, and $T_{h}$/$T_{c}$ denote the temperatures at the hot/cold side.

The power generation of the TEG is primarily influenced by the temperature gradient. In order to maximize the temperature difference between the two ends of the TEG, it is necessary to dissipate the heat on the cold side of the device [33]. This is particularly important given the high thermal conductivity of TEGs. Table 3 presents the different types of heat sinks commonly used in thermal energy-harvesting systems.

In the context of miniaturizing WSN nodes, a semi-active heat sink is an ideal solution that balances power dissipation capacity, surface area, and volume. However, a single TEG may not efficiently harvest thermal energy. To address this, two TEGs were vertically stacked and outputted in series to boost the energy output. Given the high thermal conductivity of TEGs, there is often thermal energy overflow from the cold side of a single TEG. By vertically stacking a second TEG, this overflow energy could be harvested, maximizing the energy efficiency. To reduce thermal resistance and minimize energy loss, each device’s contact surface was coated with thermal conductive silicone grease.

To effectively harvest energy from multiple energy sources, a multi-source energy-harvesting strategy is required. Generally, such a strategy can be designed in one of two ways:

1. Connect each energy harvester in series or parallel to a DC/DC converter for energy harvesting. While this approach enables one DC/DC converter to be used for two power sources, it requires that the power sources have similar internal impedance [35]. In our system, two TEGs were connected in series, resulting in an internal resistance of 12 Ω, which was much lower than the kΩ-level internal resistance of the solar cell.
2. Alternatively, each energy harvester can be connected to a separate DC/DC converter, which is then connected in series with a diode and finally outputted in parallel (also known as power OR-ing) [36]. While this method may result in some energy loss, each energy harvester can be highly efficient at harvesting ambient energy. This strategy is particularly suitable for hybrid energy harvesting of thermal and solar energy, as in our system. Notably, both the ADP5090 and MCRY12-125Q-46WI chips have a diode built into the output, obviating the need for extra diodes.

### 3.2. Hybrid Energy Storage Subsystem

Energy management circuits are responsible for storing excess energy in the battery when ambient energy is sufficient and for supplying continuous power from the battery when ambient energy is insufficient. In practice, sensing nodes have a pulsed power demand, requiring the use of SC to absorb the power pulses. However, the strategy of energy distribution between the battery and SC needs to be designed.

**Figure 4:**
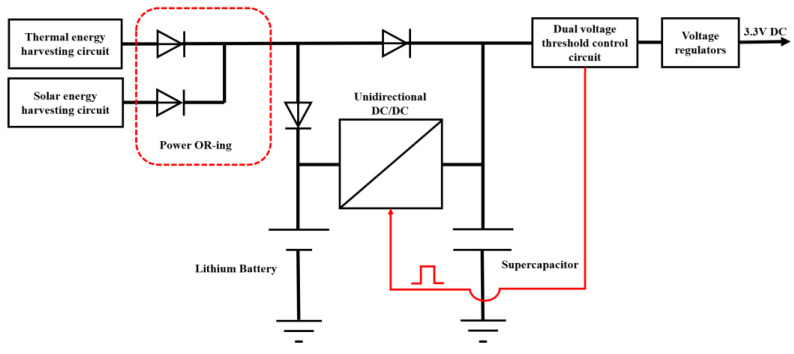
Schematic diagram of the hybrid energy storage subsystem.

In this article, a hybrid energy storage subsystem consisting of an energy management module, a battery, and an SC was utilized, as shown in Figure 4. The energy management module employed a hybrid diode semi-active topology, and the energy distribution method was based on a modified SC first EMS.

The battery and SC in self-powered IoT nodes were connected by a unidirectional DC/DC. The diode directly connected to the DC/DC was used to isolate the SC from the battery, thus preventing the SC voltage from being clamped. This hybrid diode semi-active topology aimed to store ambient energy in the battery and compensate for generation–demand mismatch with the SC. The dual voltage threshold control circuit monitored the voltage of the SC and controlled the unidirectional DC/DC on and off based on the SC threshold range. The energy management module used the SC as an energy buffer to directly supply power to the load, while the battery indirectly provided power to meet the average power consumption of the nodes. This topology helped to avoid high current discharge of the battery and prolonged the system’s lifetime.

As a load model for an RFID tag-based IoT sensor node, consider an operating voltage of 3.3 V, transmit power of 1 dBm, operating frequency of 2.4 GHz, sleep mode current of 1 μA, and an operating state peak current of about 20 mA. If the sensor node is set to transmit every 300 ms with an average current of about 70.46 μA, the average power consumption of the sensor node will not exceed $P_{average}$, as calculated by Equation (2):

(2) $$P_{average}=V\times \bar{I}$$

where $P_{average}$ is the average power of the sensor node, V is the operating voltage of the sensor node, and $\bar{I}$ is the average current of the node. $P_{average}$ is calculated to be about 232.52 µW.

The battery needs to be able to provide enough energy for the node to operate for at least 24 h in the absence of heat or light. Therefore, the battery capacity should satisfy Equation (3):

(3) $$Q_{bat}\times V_{bat}\times \eta \geq 24\times P_{average}$$

where $Q_{bat}$ is the capacity of the battery, η represents the efficiency of the unidirectional DC/DC converter, which is assumed to be 80%, and $V_{bat}$ is the maximum discharging voltage of the battery, set at 4.2 V. Based on Equation (3), it was calculated that the battery should have a capacity larger than 1.66 mAh. In order to leave some margin for the system, a small 7 mAh metal button cell was selected.

**Figure 5:**
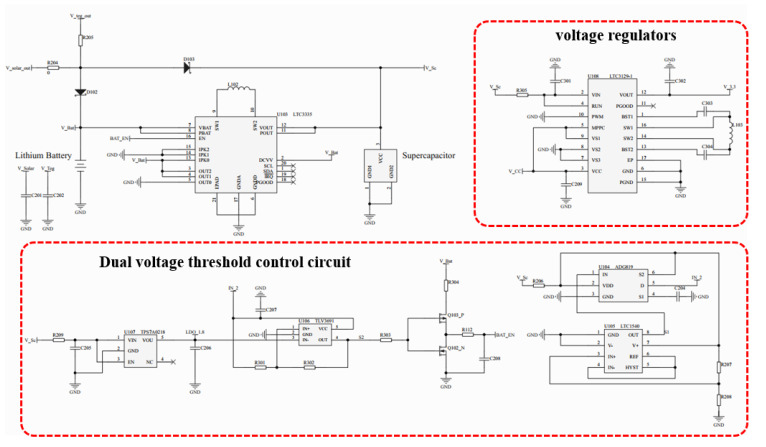
Schematic diagram of energy management circuit.

This paper presents the design of an energy management module based on a hybrid diode semi-active topology that used a 6.8 mF SC with low equivalent series resistance (ESR) and low leakage current as the power buffer. The circuit implementation is illustrated in Figure 5. The unidirectional DC/DC converter used in the design was the LTC3335, which has a quiescent current of 680 nA. This buck-boost converter has a programmable peak input current range of 5 to 250 mA, and its peak input current limit in this system was set to 10 mA to prevent high-current discharge of the battery. The dual voltage threshold control circuit was composed of low-power chips, including the LDO chip TPS7A0218, the comparator chip TLV3691, the electronic switch chip ADG819, the chip LTC1540, and non-gates (i.e., composed of PMOS and NMOS). The design also incorporated the LTC3129-1, a buck-boost converter with low quiescent current and high efficiency, which was set to output a voltage of 3.3 V. The quiescent power of the management circuit was calculated to be less than 10 µW based on the data sheet for each chip’s quiescent current and operating voltage, thus meeting the system’s demand for low power consumption.

**Figure 6:**
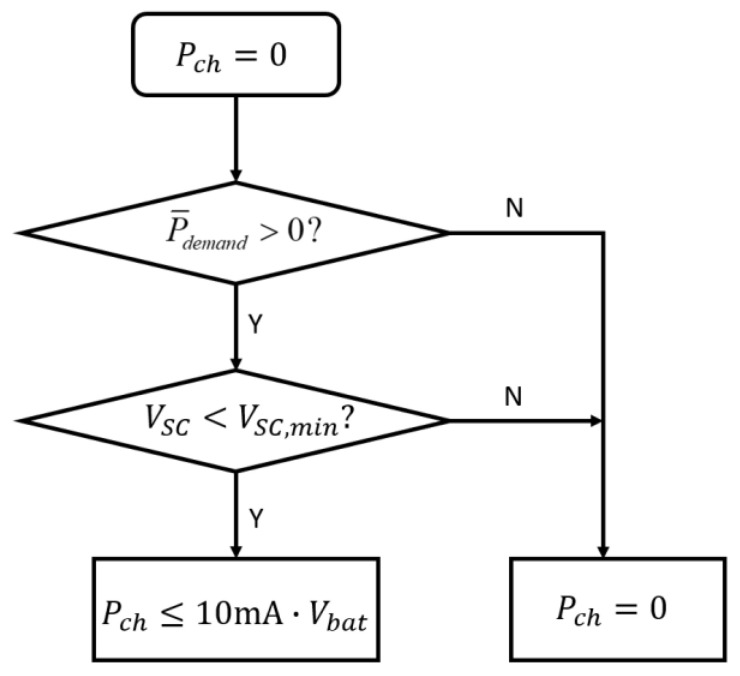
Modified SC first EMS of hybrid diode topology.

The flow chart of the modified SC first EMS is shown in Figure 6, where $V_{bat}$ is the voltage of the battery, $P_{ch}$ is the output power of the battery (if $P_{ch}$ is positive, the SC is charged by the battery; otherwise, the battery is discharged by the SC), ${\bar{P}}_{demand}$ is the system power demand, $V_{SC}$ is the voltage of the SC, and $V_{SC,min}$ is the minimum discharge voltage of the SC. The voltage range of the SC was set to be 2.5–4.2 V. When the harvested power was insufficient (${\bar{P}}_{demand}$ > 0), the battery charged the SC with no more than 10 mA current until the SC was fully charged. At this time, the discharge power of the battery was larger than the average load power, and the SC would be fully charged in a short time to respond to the variable power demand of the load. Conversely, when the harvested power was more than needed (${\bar{P}}_{demand}$ < 0), $P_{ch}$ was set to 0, and the battery or SC was charged by the energy-harvesting unit according to their respective voltages.

The hybrid energy storage method used in this system offered a fast response to the high current demand of the load when the ambient energy was insufficient. In addition, the use of hybrid energy storage significantly prolonged the node’s lifetime by avoiding high current charging/discharging of the battery and fully utilizing the SC’s capabilities.

### 3.3. System Integration

**Figure 7:**
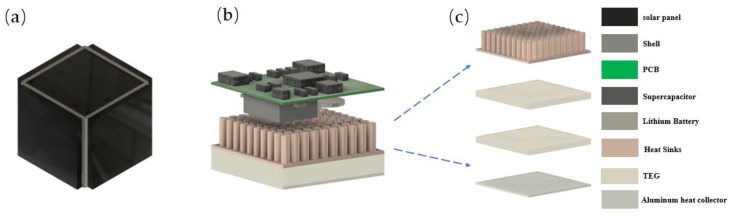
(**a**) 3D model of the self-powered sensor node; (**b**) system internal components; (**c**) the TEG device.

Figure 7a shows the 3D model of the self-powered sensor node, which had a cube shape with dimensions of 44 mm × 44 mm × 40 mm, resulting in a volume of 77.44 cm³. The system had five light-receiving surfaces, each covered with a 40 mm × 40 mm × 1.1 mm solar cell. The TEG device was located at the bottom of the system, and its internal structure is illustrated in Figure 7c. As shown in Figure 7b, the energy storage devices were mounted on the back of the PCB. The shell was manufactured using 3D printing technology, which minimized the system’s size while minimizing space redundancy and increasing the system’s energy and power density.

## 4. Energy-Harvesting Subsystem Testing

### 4.1. Multi-Sided PV Output Testing

The performance of multi-sided PVs is a complex interplay between several factors, including the intensity of light and angle of irradiation. Consequently, it is imperative to conduct a thorough investigation of the intricate interplay between light intensity, irradiation angle, and PV output power. Such an inquiry holds the potential to shed light on optimal design strategies and inform the development of more efficient and effective PV systems.

#### 4.1.1. Theoretical Derivation of Ground Projection from the System

**Figure 8:**
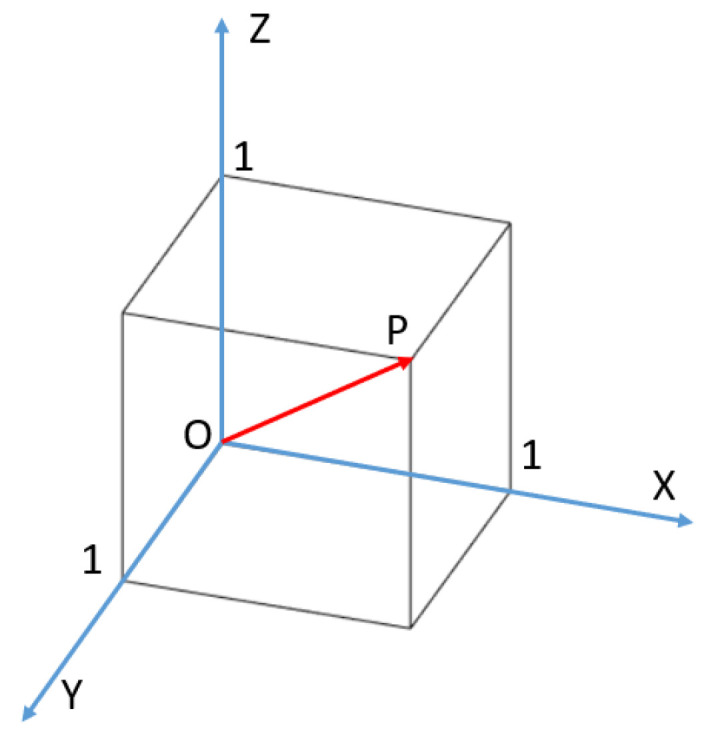
The XYZ coordinate system of the system.

The system could be approximately considered as a cube with three equal sides. The XYZ coordinate system was defined with the origin O = (0, 0, 0), as seen in Figure 8. According to Euler’s rotation theorem, any 3D rotation can be specified using two parameters: a unit vector that defines an axis of rotation and an angle *θ* describing the magnitude of the rotation about that axis [37]. The unit vector of the rotation axis is $\vec{OP}$ = (1,1,1). The objective is to rotate the $\vec{OP}$ to coincide with the *Z*-axis; at this time, the system has the biggest projection to the ground. Assuming $\vec{OP}$ is rotated α degrees around the *X*-axis and then β degrees around the *Y*-axis to coincide with the *Z*-axis, $\vec{OP}$’s coordinates at this point are (0, 0, $\sqrt{3}$) due to the fact that its length has not changed during the rotation.

According to the relationship between the rotation matrix and unit vectors, we can obtain Equation (4):

(4) $$A\;\cdot \;B\;\cdot \left[\begin{array}{c} 1\\ 1\\ 1 \end{array}\right]=\left[\begin{array}{c} 0\\ 0\\ \sqrt{3} \end{array}\right]$$

where *A* is the rotation matrix $\left[\begin{array}{ccc} cos\beta & 0 & sin\beta \\ 0 & 1 & 0\\ -sin\beta & 0 & cos\beta \end{array}\right]$ of the vector rotating around the *Y*-axis, and *B* is the rotation matrix $\left[\begin{array}{ccc} 1 & 0 & 0\\ 0 & cos\alpha & -sin\alpha \\ 0 & sin\alpha & cos\alpha \end{array}\right]$ of the vector rotating around the *X*-axis.

According to Equation (4), α = 45° and β ≈ −35°. When the system rotates *A* and *B* around the *X* and *Y* axes, respectively, the system can theoretically harvest the most energy.

#### 4.1.2. Indoor Fluorescent Lamp Experiment

**Figure 9:**
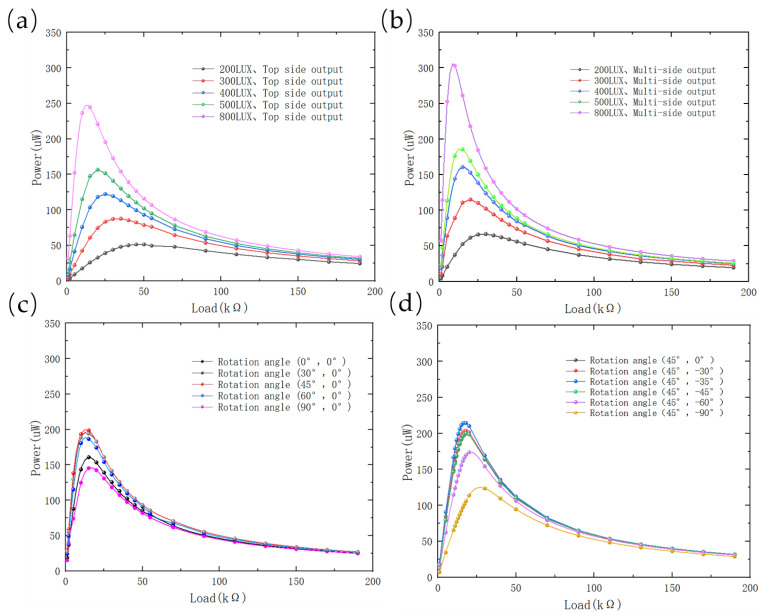
(**a**) P-R curves for single-sided PV; (**b**) P-R curves for multi-sided PVs; (**c**) P-R curves for single-sided PV with different rotation angles (fixed light intensity 400 lux, rotation around *X*-axis); (**d**) P-R curves for multi-sided PVs with different rotation angles (fixed light intensity 400 lux, rotation around *Y*-axis). The point data in the graphs were measured and the curves were fitted after three interpolations of the point data.

To examine the energy output performance of the multi-sided PVs under indoor fluorescent lamp irradiation, an experimental platform was developed to enable the variation of light intensities and adjustments to the system’s position in order to determine the effect of light on output power. The indoor test setup comprised a 22 W fluorescent lamp with a color temperature of 6000 K, a rotatable tripod head, a digital lux meter, a digital multi-meter, and a resistance box. As presented in Figure 9a, the impact of varying light intensities on the maximum power output of the top surface PV was evaluated, while Figure 9b depicts the maximum output power of multi-sided PVs at different light intensities. The results indicated that the multi-sided PVs offered a 27% higher maximum output power compared to single-sided PVs. Additionally, Figure 9c,d illustrates the maximum output power of the multi-sided PVs at various positions under a constant 400 lux light intensity. It was observed that the maximum output power was achieved when the multi-sided PVs were rotated by 45° and −35° around the *X* and *Y* axes, respectively, resulting in a remarkable 31.11% increase in the maximum output power compared to the 0° rotation. The maximum output power obtained at this position was 210.15 µW, which represented a 75.13% increase compared to the maximum output power of a single-sided PV.

**Figure 10:**
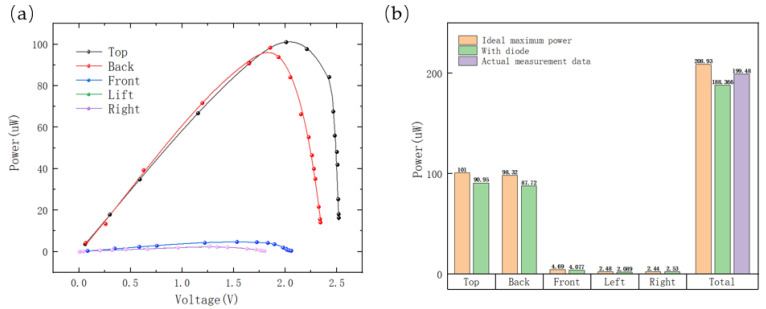
(**a**) P-V curve of a single-sided PV; (**b**) comparison of the output power of the scheme proposed in this paper with that of the traditional scheme.

Furthermore, when the multi-sided PVs were rotated by 45° around the *X*-axis, the output power and voltage of each surface PV in the system were analyzed, as illustrated in Figure 10a. It was observed that the open-circuit voltage of each surface PV did not show any significant difference. In a traditional multi-sided scheme, a series diode is added to each surface PV to prevent mutual interference between the PVs. The standard Schottky diode voltage differential ranges from 0.1 to 0.3 V. Taking into account that the harvested energy was confined to the micro-watt range, a diode voltage differential of 0.2 V was selected to calculate the maximum harvested energy by each side of the traditional multi-sided scheme, with each PV connected in series with a diode, as well as their sum. In contrast, the proposed scheme of connecting each PV directly in parallel was examined to determine the potential for enhanced energy output. The results are illustrated in Figure 10b, revealing that the total maximum output power was higher by approximately 10.92% in the proposed scheme as compared to the traditional multi-sided scheme. In experimental evaluations, the proposed method exhibited exceptional performance with the maximum harvested energy being close to the ideal maximum energy. These findings validated the high effectiveness of the proposed approach.

#### 4.1.3. Scattered Light Experiment

**Figure 11:**
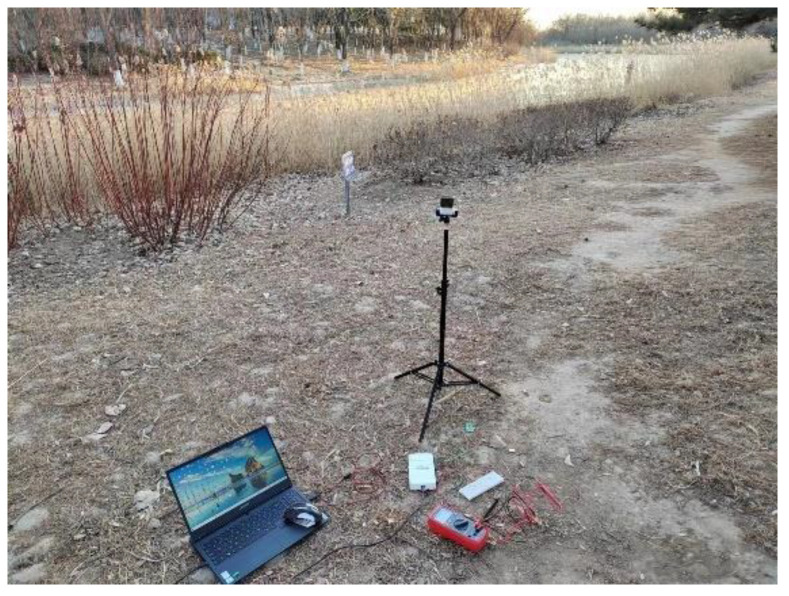
Experimental setup for scattered solar radiation experiment.

The primary objective of this experiment was to examine the output power of multi-sided PVs under various forms of scattered light, encompassing scenarios such as indoor sunlight that is scattered through windows as well as sunlight that is scattered in outdoor forested areas. In optimal conditions of scattered light, the light intensity on every surface of the multi-sided PVs was anticipated to be uniform. The experimental location chosen for this study was a forest park, as depicted in Figure 11, and the measurements were taken at 4:00 pm when the temperature was recorded as −3 °C. The light intensity values were 2311 lux on the top surface, 1602 lux on the back, 2238 lux on the front, 1920 lux on the left, and 1701 lux on the right. These values were similar, which approached the ideal conditions of scattered light.

**Figure 12:**
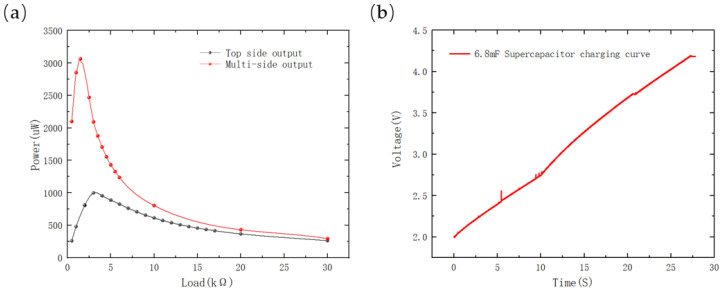
(**a**) The output power of top-sided PV and multi-sided PVs; (**b**) charging curve of a 6.8 mF SC.

The measurements of output power of a single-sided PV and multi-sided PVs are illustrated in Figure 12a. The maximum output power of the top surface PV was found to be 999.2 μW, while that of the multi-sided PVs was recorded as 3440.1 μW. This indicated that the output power of multi-sided PVs was 2.44 times higher than that of a single-sided PV. Furthermore, when the multi-sided PVs were connected to the energy-harvesting subsystem for charging an SC, the SC voltage was recorded, as depicted in Figure 12b. It was found that the 6.8 mF SC was charged to 4.2 V within approximately 27 s, and the average charging power of the capacitor was 1.72 mW. This value was deemed to be sufficient for powering many IoT sensing nodes.

### 4.2. Thermal Energy Harvesting Testing

**Figure 13:**
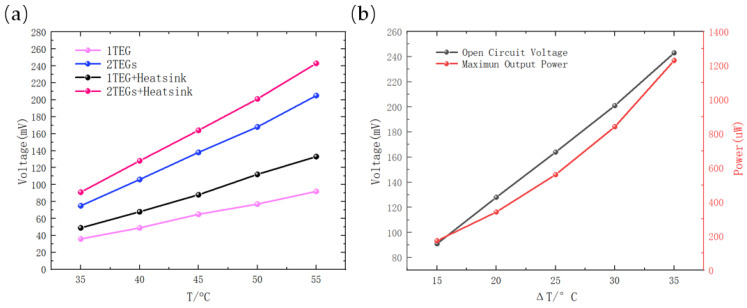
(**a**) Changes in open-circuit voltage with temperature gradient for different configurations of the TEG device; (**b**) performance of the TEG device comprising two vertically stacked TEGs and a heatsink.

In Section 2, an analysis of TEG performance revealed that stacking multiple TEGs could significantly increase the output power. To verify this finding and evaluate the performance of a TEG device under varying temperature gradients, we constructed a thermal energy harvesting testing platform. As depicted in Figure 13a, the TEG device comprised two vertically stacked TEGs and a heatsink. The experimental results showed that this TEG combination produced an open-circuit voltage approximately 2.52~2.64 times higher than that of a single TEG, with an ideal maximum output power of approximately 319.48~348.82% of a single TEG. Figure 13b illustrates the variations of the output power and open-circuit voltage of the TEG device under different temperature gradients. When the ambient temperature was 20 °C and the heating platform was set to 55 °C, the temperature difference ΔT between the heating platform and environment was measured to be 35 °C after the system reached thermal equilibrium, resulting in an ideal maximum output power of approximately 1.23 mW.

## 5. System-Level Testing and Demonstration Verification

### 5.1. System Testing

**Figure 14:**
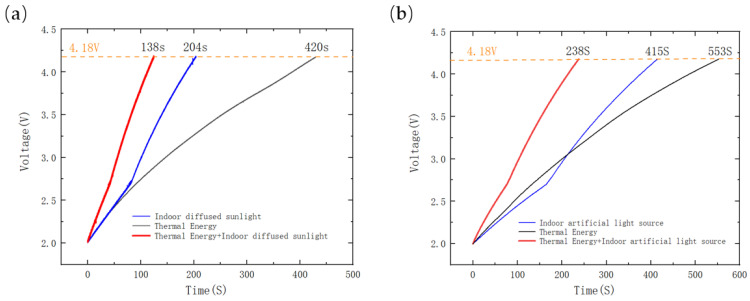
(**a**) Indoor scattered natural light test (daytime); (**b**) fluorescent light test (nighttime).

Indoor natural light testing was conducted at 4:00 p.m. with an indoor temperature of 19.1 °C, and the heating platform was set to 40 °C. The illuminance on the top surface of the system was measured to be 355 lux, and the power generated from indoor scattered natural light, thermal energy, and their combination was measured. The thermal energy was harvested by setting the heating platform to 40 °C and allowing the system to reach thermal equilibrium. The results, depicted in Figure 14a, demonstrated that the average charging power for thermal energy was approximately 106.53 µW, while for indoor scattered natural light, it was 272.66 µW. Interestingly, when thermal energy and indoor scattered natural light were combined, the average charging power increased to 369.44 µW. Compared to solar energy alone, the combination increased power harvest by 35.49%, and compared to thermal energy alone, power harvest was remarkably increased by 246.79%.

Additionally, fluorescent light testing was conducted at 7:00 p.m. when the indoor temperature was 20.9 °C, and the heating platform was set at 40 °C. The top surface illuminance of the system was measured at 364 lux. Figure 14b shows the charging curve of a 6.8 mF SC. The average charging power for thermal energy was approximately 82.84 µW, while for indoor fluorescent light, it was 110.39 µW. The average charging power for the mixture was 192.48 µW. The mixture increased power harvesting by 74.36% compared to solar energy alone and by 132.35% compared to thermal energy alone.

### 5.2. Demonstration Application

**Figure 15:**
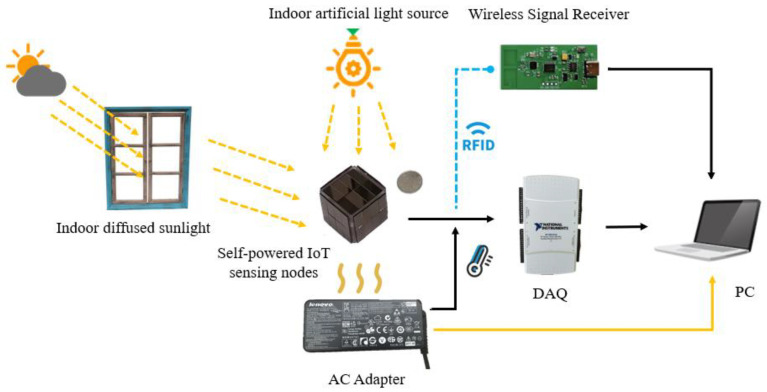
Application demonstration of temperature monitoring and early warning for indoor AC adapter.

Table 4 displays the energy consumption of the communication transceivers for RFID, ZigBee, and Wi-Fi. The selection of the RFID communication transceiver in this study was justified by the requirement for low power consumption. As a proof-of-concept trial and case study, this paper employed an RFID tag-based IoT sensor node to demonstrate the usability of the proposed self-supply method in a real-world application. The sensor node periodically reports data to the gateway node, which then connects to a host computer to display the monitoring data. After reporting, the sensor node immediately goes into sleep mode to maintain low power consumption. The experimental setup is shown in Figure 15, and the energy harvested by the node came from indoor scattered natural light during the daytime, fluorescent lamp light during the evening, and the waste heat continuously generated by the high-power computer adapter. In addition, the NI USB-6216 was used to capture the voltage and current signals of the battery and SC in the node.

**Figure 16:**
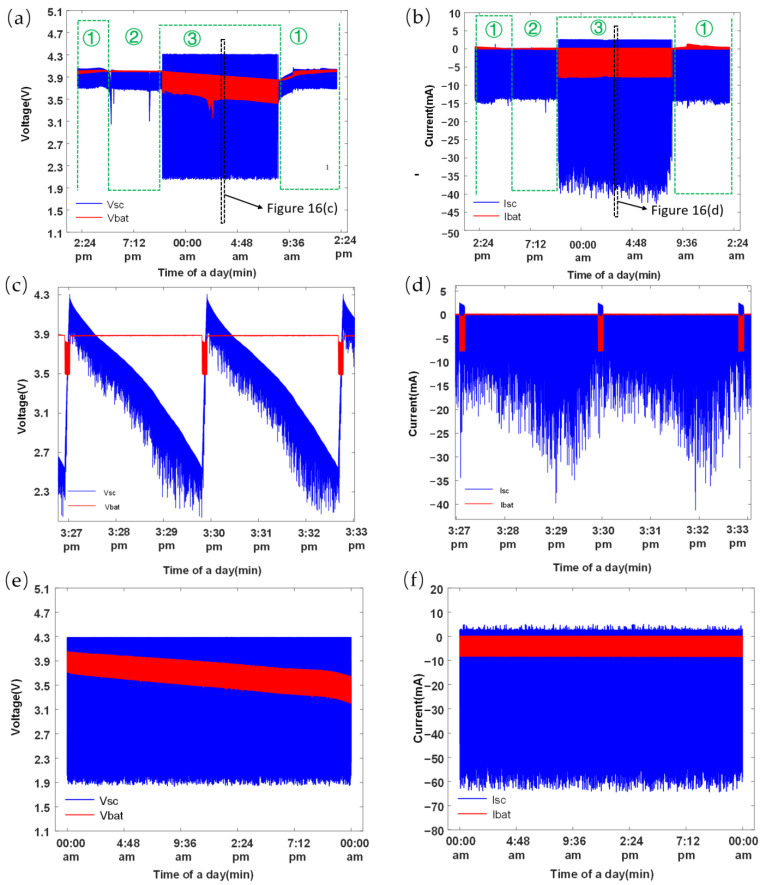
Daily performance of self-powered IoT sensor nodes. (**a**) The voltages of battery and SC; (**b**) The currents of battery and SC; (**c**) Local enlargement of (**a**); (**d**) Partial enlargement of (**b**); (**e**) The voltages of battery and SC under extreme conditions; (**f**) The currents of battery and SC under extreme conditions. The serial numbers in the sub-figure (**a**,**b**) indicate different time periods, with 1 representing 7:30 a.m. to 5:00 p.m., 2 representing 5:00 p.m. to 9:00 p.m., and 3 representing 9:00 p.m. to 7:30 a.m., covering a total of 24 h.

The self-powered IoT sensor node was used to monitor the temperature of the AC adapter for 24 h. The current and voltage changes of the battery and SC in the system were measured to show the energy flow, as depicted in Figure 16. The period from 7:30 a.m. to 5:00 p.m. had natural scattered light and waste heat from electrical appliances as energy sources. Between 5:00 p.m. and 9:00 p.m., the energy source was the heat from fluorescent lights and other appliances, while from 9:00 p.m. to 7:30 a.m., it was the heat from electrical appliances. The voltage changes of the battery and SC over a 24 h period are shown in Figure 16a. Remarkably, the results indicated that the battery voltage did not decrease from the previous day, implying that the self-powered node could operate steadily in the laboratory for an extended period. In addition, the SC was frequently discharged to power the transmit power of the node while the battery voltage remained stable, as shown in Figure 16c. As seen in Figure 16b, the battery was discharged at low current whereas the SC was discharged at high current to meet the power demand of the node. Moreover, when the voltage of the SC fell below 2.5 V, the battery charged the SC with a current of no more than 8 mA for a brief time to meet the next round of power demand from the sensor node during the transmitting state, as shown in Figure 16d. Furthermore, the system underwent testing under extreme conditions (i.e., a 24 h period without any environmental energy). Figure 16e illustrates the voltage fluctuations of the lithium battery and SC during this scenario, indicating that the lithium battery’s voltage dropped from 4.035 to 3.623 V. Despite this decrease, a substantial amount of energy still remained in the battery, given its nominal voltage of 3.6 V. Figure 16f depicts the current variations of the lithium battery and SC under these harsh conditions, demonstrating the system’s stable operation. These findings suggested that the system was reliable and robust.

After the analysis, it was evident that the system functioned as designed and could operate stably for a long time in an indoor environment where both light and thermal energy were present.

### 5.3. Comparison with Related Works

Recently published energy-harvesting systems are presented in the literature. The proposed design was compared with existing energy-harvesting systems for WSNs or IoT, and the results are illustrated in Table 1. The proposed energy-harvesting system achieved a utilization rate of up to 100% for the light-receiving surface area of the system, which represents a significant improvement compared to traditional indoor light energy-harvesting systems. This improvement enabled the IoT nodes to harvest sufficient energy even under indoor lighting conditions. Additionally, the proposed hybrid energy-harvesting system provided more reliable electricity in diverse application scenarios compared to a single energy source harvester. The system also incorporated a hybrid energy storage subsystem that employed a hybrid diode semi-active topology, enabling efficient energy management. The entire system was compactly integrated into a 3D printed box. However, the existing system has a rectangular cuboid shape that may not be suitable for situations with specific shape requirements. Furthermore, the amount of harvested energy is still limited. To advance this technology, the incorporation of flexible energy-harvesting devices could be explored to enable the system to adapt to any shape. Additionally, using high conversion efficiency PV cells and TEGs could result in increased energy output.

## 6. Conclusions

This article presents a solution for self-powered IoT sensor nodes in indoor environments by proposing a hybrid energy-harvesting system that harvests indoor light and thermal energy. The system was integrated with IoT sensor nodes in a 3D printed box. The system included five light-receiving surfaces equipped with PVs to harvest indoor light energy, while a TEG device was installed at the bottom to harvest heat energy. Under fluorescent lamps and scattered natural light, the maximum energy harvested by the multi-sided PVs was increased by 75.13% and 244.29%, respectively, compared to a single-sided PV. By connecting each PV directly in parallel, the maximum harvested power was increased by 10.92% compared to the traditional multi-sided scheme with each PV connected in series with a diode. The TEG device, composed of two vertically stacked TEGs and a radiator, had an open-circuit voltage about 2.52 times greater than that of a single TEG and could harvest waste heat under lower temperature differences. A hybrid energy storage subsystem was designed to manage the harvested energy. The system was tested in the presence of continuous waste heat. It achieved a mixed average charging power of 192.48 µW under fluorescent lamp illumination and 369.44 µW under indoor scattered natural light. The self-powered sensor node was successfully implemented and evaluated for high-power appliance monitoring and warning applications in indoor environments where both light and thermal energy are present, providing an effective solution for powering WSN nodes.

## Figures and Tables

**Table 1:** Related work in recent years.

Refs	System Integrated in a Package	Indoor or Outdoor	Energy Sources	Area (PV Cell)/Area (System Lighted Surface)	Number of TEGs	Hybrid Energy Storage Topology	Year
[26]	Yes	outdoor	PV, PZT	<50%	-	Passive	2014
[17]	No	Indoor	PV	<50%	-	-	2017
[18]	No	Indoor	PV, TEG	<20%	Single	-	2021
[27]	No	outdoor	PV	<80%	-	Semi-active	2021
[19]	Yes	outdoor	PV	100%	-	-	2022
This work	Yes	Indoor	PV, TEG	100%	Two	Semi-active	-

**Table 2:** The power produced from indoor ambient sources [29].

Energy Source	Harvesting Device	Power Density	Harvested Power
Indoor Light	Solar Cell	0.1 mW/cm^2^	10 µW/cm^2^
Outdoor Light		100 mW/cm^2^	10 mW/cm^2^
Human Thermal	Thermoelectric Generator	20 mW/cm^2^	30 µW/cm^2^
Industrial Thermal		100 mW/cm^2^	1–10 mW/cm^2^
RF: GSM 900 Mhz	Antenna	0.3 µW/cm^2^	0.1 µW/cm^2^
RF: Wi-Fi		0.015 µW/cm^2^	0.001 µW/cm^2^

**Table 3:** Classification of heat sink groups [34].

Heat Sink Category	Advantages	Disadvantages	Examples
Passive	User friendly Readily available Cheap	Low power dissipation	Metal plate
Semi-active	Low thermal resistance	Low power dissipation	Fin heat sink
Active	Low thermal resistance High heat dissipation	Low long-term reliability High cost	Fan-fins heat sink

**Table 4:** Energy consumption of the RFID, ZigBee, and Wi-Fi communication transceivers.

Type	Operating Voltage	Sleep Mode Current	Operating Mode Peak Current
RFID	3.3 V	1 µA	20 mA
ZigBee [38]	3.3 V	10.1 µA	59.65 mA
Wi-Fi [39]	5 V	18.93 mA	155.17 mA

## Data Availability

Not applicable.
